# Non-fluent/Agrammatic Variant of Primary Progressive Aphasia With Generalized Auditory Agnosia

**DOI:** 10.3389/fneur.2020.00519

**Published:** 2020-06-26

**Authors:** Hiroyuki Watanabe, Manabu Ikeda, Etsuro Mori

**Affiliations:** ^1^Department of Behavioral Neurology and Neuropsychiatry, United Graduate School of Child Development, Osaka University, Suita, Japan; ^2^Department of Psychiatry, Graduate School of Medicine, Osaka University, Suita, Japan; ^3^Brain Function Center, Nippon Life Hospital, Osaka, Japan

**Keywords:** agrammatism, amusia, apraxia of speech, environmental sound agnosia, word deafness

## Abstract

Cortical neurodegeneration-induced non-fluent/agrammatic variant of primary progressive aphasia (nfvPPA) is a clinical syndrome characterized by non-fluent speech, such as apraxia of speech or agrammatism. We describe the case of an 80-year-old right-handed woman who exhibited nfvPPA. Atypically, our patient also presented with generalized auditory agnosia. Brain magnetic resonance imaging revealed left-sided predominant atrophy of the bilateral perisylvian area, including the inferior frontal and superior temporal lobes. In a series of auditory tasks assessing generalized auditory agnosia, our patient was unable to accurately identify verbal sounds, environmental sounds, or familiar Japanese songs that she could sing. In the context of recent studies, our study indicates the existence of a clinical syndrome characterized by progressive speech disorder with auditory agnosia. This case report thus provides novel insights into the spectrum of language impairment induced by neurodegenerative disease.

## Background

Primary progressive aphasia (PPA) is a collective term for neurodegenerative diseases that present with language impairment as the most salient feature. Consensus criteria were proposed in 2011 for three clinical syndromic variants of PPA: non-fluent/agrammatic (nfvPPA), semantic, and logopenic (1). nfvPPA is characterized by non-fluent speech, such as apraxia of speech (AOS) or agrammatism; the semantic variant of PPA, by anomia with loss of the meanings of single words; and the logopenic variant of PPA, by anomia without loss of the meanings of single words, sentence repetition deficits, and phonological errors. However, recent evidence suggests the existence of an additional, atypical variant of PPA (2–4), indicating that the established consensus criteria may not account for the full range of clinical syndromic variants of PPA. Herein, we present the case of a patient with nfvPPA and generalized auditory agnosia to further expand our knowledge of the spectrum of language impairment in neurodegenerative diseases.

## Case Presentation

### Case Description

**Figure 1:**
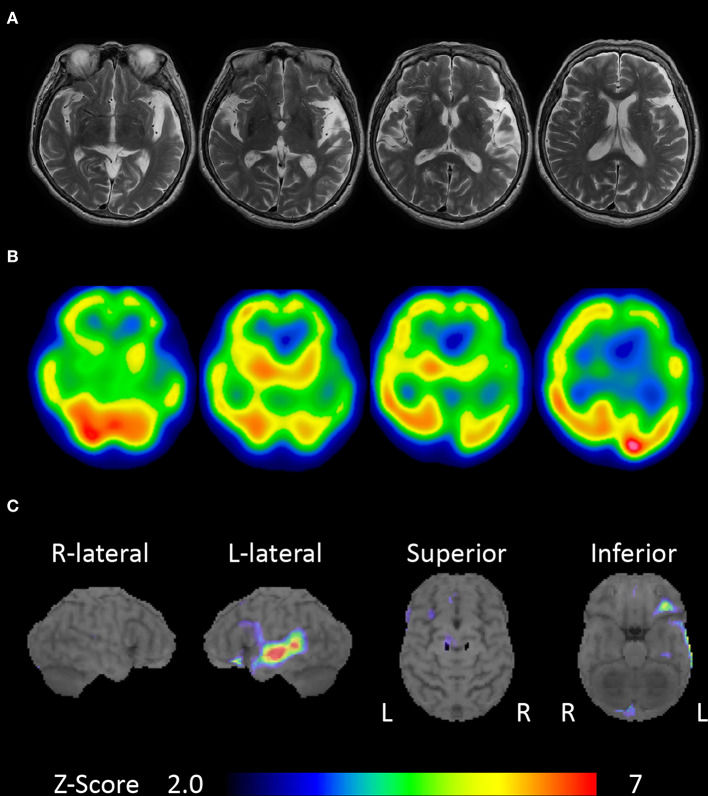
Brain magnetic resonance imaging and single-photon emission computed tomography (SPECT). **(A)** Brain magnetic resonance imaging showing left-sided predominant atrophy of the perisylvian area. **(B)** *N*-Iso-propyl-*p*-[123I] iodoamphetamine SPECT showing left-sided predominant hypoperfusion of the bilateral frontal and temporal lobes. **(C)** Brain SPECT analyzed with 3D stereotactic surface projections (SSPs) showing relative hypoperfusion mainly in the left superior temporal and inferior frontal gyri.

An 80-year-old, right-handed woman visited our hospital because of gradually progressive difficulty in speaking and recognizing spoken words. She had received 9 years of education. Speaking and recognizing spoken words had concurrently become challenging at around the age of 77 years. Except for the presence of cataract, her medical history was unremarkable. She was fully conscious and oriented at the initial visit. No abnormalities were detected on physical and neurological examinations, or routine laboratory tests. Brain magnetic resonance imaging revealed left-sided predominant atrophy of the bilateral perisylvian area (Figure 1A). There was no evidence of hemorrhage or ischemic lesion. *N*-Iso-propyl-*p*-[123I] iodoamphetamine single-photon emission computed tomography (SPECT) revealed predominant left-sided hypoperfusion of the bilateral frontal and temporal lobes (Figure 1B). To assess the patterns of hypoperfusion (5), SPECT data were analyzed with 3D stereotactic surface projections (SSP) (6). All SPECT scans underwent realignment, spatial normalization, and non-linear warping. The scans were sampled at 16,000 predefined cortical locations and projected on a 3D image. The voxel values of the patient's SPECT data were normalized to the whole brain's tracer uptake and compared with an age-matched normal database, yielding a 3D SSP *Z* score image. The abnormalities of cerebral hypoperfusion were displayed with a *Z* score map. *Z* scores were calculated using the following equation: *Z* score = (normal mean – patient mean) / (normal standard deviation). We used a *Z* score of 2 as the cutoff value in each voxel, and voxels with a *Z* score ≤ 2 were considered voxels without significantly decreased regional cerebral blood flow. Brain SPECT data analyzed with 3D SSP revealed relative hypoperfusion, mainly in the left superior temporal and inferior frontal gyri (Figure 1C).

### Neuropsychological Examination

Detailed neuropsychological evaluations were performed in the month following the initial visit. Detailed data obtained from standard neuropsychological tests are presented in Table 1. The Wechsler Adult Intelligence Scale—Third Edition (WAIS-III) with written instructions (7) revealed a full-scale intelligence quotient (IQ) of 98, verbal IQ of 84, and a performance IQ of 116. Using the Wechsler Memory Scale—Revised with written instructions (7), we found a general memory index of 88, verbal memory index of 80, visual memory index of 107, delayed recall index of 84, and attention/concentration index of 92. These findings indicated that the patient's intelligence and memory were normal.

**Table 1:** Performance on standard neuropsychological tests.

	**Score**	**Normative data; mean (SD)**
**WAB**		
Aphasia quotient (100)	49.8	97.7 (3.0)
Fluency (10)	5	10.0 (0)
Information content (10)	8	9.7 (0.6)
Auditory comprehension (10)	5.2	9.8 (0.1)
Repetition (10)	0.4	9.9 (0.3)
Naming (10)	6.3	9.5 (0.6)
Reading (10)	9.1	9.5 (0.8)
Writing (10)	7.1	9.6 (1.0)
Praxis (60)	57	59.8 (0.7)
Calculation (24)	24	23.1 (2.3)
**Token test**		
Auditory comprehension (166)	6	163.6 (2.0)
Reading (166)	166	164.8 (1.5)
**WAIS-III**		
Full IQ	98	100.0 (15.0)
Verbal IQ	84	100.0 (15.0)
Performance IQ	116	100.0 (15.0)
**Raven's colored matrices** (36)	32	24.9 (5.3)
**WMS-R**		
General memory index	88	100.0 (15.0)
Verbal memory index	80	100.0 (15.0)
Visual memory index	107	100.0 (15.0)
Attention/concentration index	92	100.0 (15.0)
Delayed recall index	84	100.0 (15.0)

*The maximum score is noted in each row header*.

*WAB, Western Aphasia Battery; WAIS-III, Wechsler Adult Intelligence Scale—Third Edition; IQ, intelligence quotient; WMS-R, Wechsler Memory Scale—Revised; SD, standard deviation*.

Her spontaneous speech was shown to be monotonous, slow, and effortful using the Western Aphasia Battery [Japanese edition; (8)]. Her articulation was impaired due to AOS. Connections between syllables were frequently prolonged. She sometimes exhibited distorted sound substitutions and stuttering without self-correction. We occasionally observed telegraphic speech characterized by the omission of grammatical morphemes, which is a component of agrammatism. She experienced difficulty in producing sentences, and her speech was limited mainly to short utterances. She recognized spoken words with difficulty; hence, repetition and auditory comprehension were impaired. Moreover, she could not write words to dictation. However, the Token test with written questions demonstrated that her comprehension of written language was completely preserved (166/166; the mean score in four age-matched healthy controls at our hospital was 164.8 ± 1.5). She could correctly write what she wanted to say in both Kana and Kanji. Except for buccofacial apraxia, praxis was intact. No acalculia was noted.

### Special Assessments for Auditory Agnosia

The following special assessments were administered with written instructions.

#### Pure Tone Audiometry and Speech Audiometry Test

Slight sensorineural hearing loss was detected (43.8 dB in the right ear and 41.3 dB in the left) with a standard pure tone threshold audiometry test (Table 2). On the other hand, a speech audiometry test consisting of monosyllabic sounds showed discrimination of 0% at 10–90 dB for both ears (Table 2), although registration of pure tones was mostly preserved. These results revealed that our patient had severe word deafness.

**Table 2:** Performance on auditory tests.

	**Score**	**Normative data; mean (SD)**
Pure tone threshold	R 43.8 dB	
	L 41.3 dB	
Speech audiometry	Both 0% (10–90 dB)	
Click fusion	500 ms	1–3 ms
Click counting	2	9–11 counts
Recognition of environmental sounds (20)	7	20.0 (0)
Recognition of familiar Japanese songs (20)	11	20.0 (0)

*The maximum score is noted in each row header*.

*SD, standard deviation*.

#### Temporal Auditory Acuity Measures

To examine the temporal resolution of the auditory system, click fusion, and counting tests were performed following the method used by Albert and Bear (9). In the click fusion test, intervals between two brief binaural pulses were varied, and the patient was asked to report whether she heard one or two clicks. Normal controls can distinguish two clicks presented at 1–3-ms intervals (10); however, our patient could not distinguish clicks presented at intervals of 400 ms according to ascending and descending limits (Table 2). In the click-counting test, the patient was asked to count the number of clicks presented in 1 s. While the number of clicks countable by normal controls in 1 s ranges from 9 to 11 (11), our patient's count was inaccurate at rates of >2 clicks/s (Table 2). These results revealed that the temporal auditory resolution of our patient was severely impaired.

#### Recognition of Environmental Sounds

We assessed our patient's ability to recognize non-verbal sounds. Twenty environmental audio recordings consisting of the following four sound categories were presented to both ears: human non-verbal (e.g., baby crying), manmade inanimate (e.g., running water), non-human animate (e.g., dog barking), and natural inanimate (e.g., wind) (12, 13). After hearing each sound, she was asked to name the environmental sound. While the mean score of four age-matched healthy controls from our hospital was 18.0 ± 0.7, our patient could name only 2 of the 20 (10%) sounds. After the naming task, the patient was asked to match one of the four pictures to a presented sound (12, 13). The four controls easily identified the correct answers and achieved a common score of 20.0. Our patient provided correct responses for 7 of the 20 (35%) sounds (Table 2); as an example, she selected a picture of a vacuum cleaner when the sound of a ringing phone was played. These results revealed that our patient had environmental sound agnosia.

#### Recognition of Familiar Japanese Songs

The patient was asked to sing 20 familiar Japanese songs without accompaniment but with the provision of the song title and lyrics in writing; a correct response was noted when the patient's singing preserved most of the original melody. The singing score of four age-matched healthy controls from our hospital was 16.0 ± 1.6. Our patient provided correct responses for 17 out of 20 (85%) songs; her memory of the 20 songs thus seemed to have been intact. We then presented each of the songs, and the patient was asked to name the song's title or artist. While the mean naming score of the four healthy controls was 14.0 ± 1.4, our patient could identify the title and artist for only 1 out of the 20 (5%) songs. Finally, each of the 20 songs was presented to the patient, and she was asked to match the album cover, written song title, and artist name with the song in a four-alternative forced-choice paradigm. The controls were easily able to choose the correct answers and achieved a common score of 20.0. Our patient provided correct responses for 11 out of the 20 (55%) songs (Table 2). Although her singing of the familiar Japanese songs was well-preserved, she was largely unable to recognize the same Japanese songs after hearing them. These results evinced receptive amusia.

## Discussion

Herein, we present a case of unclassifiable PPA: a combination of nfvPPA and generalized auditory agnosia. The patient's speech fluency was impaired due to AOS and agrammatism, the core features of nfvPPA. She did not exhibit any problem with object knowledge as indicated by the WAIS-III score, which further supported a diagnosis of nfvPPA. In addition, neuropsychological examination revealed that she did not exhibit any problems other than conversation. Hence, except for the generalized auditory agnosia, this patient met all the criteria for nfvPPA (1).

The anterior components of the language network, including the inferior frontal lobe, and the anterior opercular and perisylvian areas, including the anterior insula and superior temporal gyrus, have been implicated as the neuroanatomical substrates of nfvPPA (14). The lesions identified in our case correspond to these areas and may therefore account for the observed language impairments, including generalized auditory agnosia, which is seldom observed in typical nfvPPA.

Generalized auditory agnosia refers to a rare impairment in the ability to recognize sounds despite adequate hearing ability, as measured using standard audiometry (15, 16). On the other hand, selective auditory agnosias refer to impairments in the ability to recognize specific categories of sounds. For example, pure-word deafness and non-verbal auditory agnosia of environmental sounds or music are considered to be selective auditory agnosias. Our patient exhibited severe word deafness despite adequate hearing ability. Her impaired temporal auditory acuity, revealed by the click fusion and counting tests, indicated the diagnosis of word deafness, which has been observed in previous patients (9, 11, 17–19). Moreover, our patient discriminated environmental sounds with difficulty and could not recognize familiar Japanese songs, even though her ability to sing those songs was well-preserved. Therefore, these results revealed that our patient had generalized auditory agnosia.

Generalized auditory agnosia, as reported in cases of cerebrovascular disease (20) and neurodegenerative disease (7, 16, 21), is associated with bilateral temporal lobe lesions involving the primary auditory and auditory association cortices; our patient's lesions, identified using brain magnetic resonance imaging and SPECT, correspond to these previously elucidated areas. Moreover, brain SPECT analyzed with 3D SSP revealed relative hypoperfusion mainly in the left superior temporal gyrus, which is consistent with patterns of hypometabolism identified using positron emission tomography with ^18^F-labeled 2-fluoro-2- deoxyglucose (7). Therefore, in the context of past research, we suspect that generalized auditory agnosia in our case was induced by bilateral temporal lobe atrophy involving the superior temporal gyrus.

Both verbal auditory (word deafness) and non-verbal auditory agnosia have been reported in the stroke literature (20) but are rarely reported in the setting of progressive neurological disorders (7). Recent evidence suggests the existence of a clinical syndrome characterized by progressive speech disorder and auditory agnosia in progressive neurological disorders: Iizuka et al. reported the case of a patient with AOS and word deafness (13); Kaga et al. described a case of AOS, word deafness, and environmental sound agnosia (22); Otsuki et al. observed the concurrent presentation of dysprosody, word deafness, and environmental sound agnosia (18); Ota et al. reported the case of a patient with progressive foreign accent syndrome and word deafness (19); Sakurai et al. described the case of a patient with unclassifiable PPA who exhibited paragrammatism, recurrent utterance, and word deafness (23); and Kuramoto et al. described the case of a patient with unclassifiable PPA with undifferentiated jargon, word deafness, and environmental sound agnosia (24). Furthermore, Utianski et al. reported the case of a patient with unclassifiable PPA who exhibited phonological errors and agrammatism of spoken and written language on first assessment (5 years after symptom onset) (7); the same patient subsequently exhibited worsening of aphasia and developed AOS as well as verbal auditory (word deafness) and non-verbal auditory agnosias. Furthermore, Mesulam et al. documented the case of a patient with unclassifiable PPA who exhibited agrammatism of spoken and written language as well as profound impaired auditory word comprehension relative to her visual word comprehension (25); the dissociation of her comprehension between auditory and visual word processing was speculatively attributed to auditory word-form area dysfunction because she could discriminate phonemes—i.e., her impairment of auditory word processing level differed from that of our patient. The other cognitive functions of the patients presented in these cases were well-preserved. The patient described by Iizuka et al. (13) subsequently developed behavioral problems. Our patient exhibited speech disorder, aphasia, word deafness, environmental sound agnosia, and receptive amusia. She did not exhibit any other cognitive impairment or behavioral problems. Moreover, recent studies have shown that patients with nfvPPA show deficits of non-linguistic auditory analysis (26, 27). However, we could not find any reports of patients with early-stage neurodegenerative diseases and unclassifiable PPA that exhibited nfvPPA and generalized auditory agnosia. To the best of our knowledge, no prior studies have involved extensive auditory examinations of a patient with nfvPPA and generalized auditory agnosia. The present case report, therefore, suggests the existence of a clinical syndrome characterized by progressive speech disorders and auditory agnosia and provides novel insight into the spectrum of language impairment induced by neurodegenerative disease.

The present study has several limitations. First, although we believed that the auditory agnosia was in the context of language deficits (7), the presence of cognitive impairments (auditory agnosia) other than aphasia may exclude a PPA diagnosis (1). Second, our patient did not undergo additional tests, other than the Western Aphasia Battery, to assess writing ability or further formal evaluation, such as the measurement of auditory-evoked potentials, to assess sensory functioning. Third, we did not perform a cerebrospinal fluid biomarker analysis. Moreover, no pathological findings were obtained in the present case, and therefore, this issue requires further investigation.

## Conclusion

The current study describes a rare case of unclassifiable PPA: a combination of nfvPPA and generalized auditory agnosia caused by neurodegenerative disease. In extensive auditory examinations assessing generalized auditory agnosia, our patient was unable to accurately identify both speech and non-speech sounds. Our results provide novel insights into the spectrum of language impairment induced by neurodegenerative disease.

## Data Availability Statement

All data generated in this study are included in the article/supplementary material.

## Ethics Statement

Written informed consent was obtained from the patient and her family members for publication of this case report and any accompanying images.

## Author Contributions

HW acquired case data, designed the study, and drafted the manuscript. MI and EM supervised the study and helped to draft the manuscript. All authors contributed to the article and approved the submitted version.

## Conflict of Interest

The authors declare that the research was conducted in the absence of any commercial or financial relationships that could be construed as a potential conflict of interest.
